# Sample adequacy in bronchoscopic ROSE: comparison between laboratory specialist and pathologist

**DOI:** 10.3389/pore.2026.1612311

**Published:** 2026-02-03

**Authors:** Gergő Szűcs, Judit Pápay, Eszter Regős, Ildikó Krencz, Márton Sághi, Péter Horváth

**Affiliations:** 1 Department of Pulmonology, Semmelweis University, Budapest, Hungary; 2 Department of Pathology and Experimental Cancer Research, Semmelweis University, Budapest, Hungary

**Keywords:** bronchoscopy, EBUS, laboratory specialist, pathologist, ROSE

## Abstract

Rapid on-site evaluation (ROSE) during bronchoscopy provides the opportunity to assess the adequacy of the sampling site, thus enabling diagnosis. The rapid evaluation of smears is typically performed by cytopathologists, but this is not always feasible. At our institution, a university-trained clinical biochemist performs the evaluation of ROSE smears. Our aim was to compare ROSE evaluations conducted by a laboratory specialist and a cytopathologist. We assessed the adequacy of lymph node samples from 78 patients using ROSE: 37 samples were obtained via EBUS-TBNA and 41 via TBNA. All smears were examined by the laboratory specialist, and simultaneously prepared parallel smears from the same needle passes were sent to the Department of Pathology for evaluation. Of the 78 samples, both the laboratory specialist and the pathologist team deemed 63 samples adequate and 8 samples inadequate. In 7 cases, there was a disagreement between the pathologist team and laboratory specialist. This resulted in a 92.3% agreement. The Cohen’s kappa value was 0.71, indicating strong and Gwet’s AC1 value was 0.90 corresponding to almost perfect agreement. The diagnostic performance was also excellent. Our conclusion is that ROSE performed by a laboratory specialist is a suitable alternative to on-site evaluation by a pathologist. It may help to overcome the resource shortage of interventional pulmonologists and cytopathologists.

## Introduction

Rapid on-site evaluation (ROSE) during endoscopic procedures, such as bronchoscopy, plays an important role in clinical practice. It saves time and cost, and reduces the need for repeated procedures due to inadequate sampling [1]. Cytological smears prepared during bronchoscopy can be assessed within minutes, allowing immediate feedback on sample adequacy. This enables the bronchoscopist to confirm or adjust the biopsy site as needed [2].

Endobronchial ultrasound-guided transbronchial needle aspiration (EBUS-TBNA) has become a reliable and indispensable diagnostic modality in the evaluation of lung cancer [3, 4]. Pathological work-up includes hematoxylin–eosin and immuncytology staining to determine the histologic subtype, while modern molecular techniques such as next-generation sequencing (NGS) allow simultaneous interrogation of multiple genes, facilitating precision oncology and treatment planning [5]. A major limitation in this pathway, however, is the availability of adequate tissue, particularly in hard-to-biopsy thoracic malignancies. Both EBUS-TBNA and conventional TBNA are cornerstone procedures in interventional pulmonology, yielding smears and cell blocks that are subsequently used by pathologists and molecular biologists for diagnosis and biomarker testing [6].

The presence of a cytopathologist at the time of sampling has been shown to improve diagnostic yield by assessing adequacy. However, due to increasing workload, this is rarely feasible in routine practice. Some studies have suggested that trained interventional pulmonologists can perform ROSE to assist adequacy assessment and thereby improve diagnostic yield [7–9]. Nevertheless, the workload of pulmonologists is already high, and it is often logistically impractical for the same operator to both perform the procedure and simultaneously evaluate ROSE, or to require a second pulmonologist solely for this task.

In our study, ROSE was performed by a university-trained clinical biochemist during bronchoscopic procedures. Our aim was to assess the accuracy of laboratory specialist–performed ROSE and to compare its results with the final cytopathologist diagnosis.

## Materials and methods

We analyzed 78 consecutive bronchoscopic sampling cases in which rapid on-site evaluation (ROSE) was performed by a university-trained clinical biochemist (laboratory specialist). Baseline characteristics are shown in Table 1. Cytological adequacy and final diagnosis were subsequently established by a board-certified team of cytopathologist, who served as the reference standard (a four-person team of pathologists).

**TABLE 1:** Baseline characteristics of the study cohort (N = 78).

Characteristic	Value
Age, mean ± SD (years)	59.4 ± 13.9
Age range (years)	27.3–82.9
Sex	
Male, n (%)	40 (51.3%)
Female, n (%)	38 (48.7%)
Procedure type	
EBUS-TBNA, n (%)	37 (47.4%)
cTBNA, n (%)	41 (52.6%)
Malignancy present, n (%)^a^	28 (35.9%)
Inflammation, n (%)^b^	19 (24.3%)

^a^ Malignancy was defined according to final cytopathologist diagnosis.

^b^ Inflammation includes sarcoidosis, granuloma, tuberculosis, abscess.

Two sampling techniques were included: endobronchial ultrasound–guided transbronchial needle aspiration (EBUS-TBNA, n = 37) and conventional TBNA (cTBNA, n = 41). Demographic data were recorded, including patient age and sex.

The samples taken during the procedures were smeared onto glass slides (1 puncture was used to obtain 2 slides). One slide was sent fresh to pathology for analysis, while the other slide was air-dried, fixed with methanol, stained with Diff-Quik rapid stain, and assessed for suitability using a light microscope (Motic 2820).

### Outcomes

1. Sample adequacy was assessed independently by the laboratory specialist (ROSE) and the pathologist team (final report). Adequacy was defined as the presence of diagnostic material (lymphocytes, tumor cells, granulomas).
2. Final diagnosis was recorded in both reports (ROSE and pathologist team) as categorical variables (“malignant lymph node”, “lymph node”, “no lymphatic elements”, “partially lymph node”, “lymph node with inflammation”).

### Statistical analysis

Descriptive statistics were used to summarize patient and procedural characteristics. For the analysis of technical adequacy, binomial proportions with Wilson 95% confidence intervals were calculated, and differences between EBUS-TBNA and cTBNA were assessed using Fisher’s exact test. Agreement between the laboratory specialist and the pathologist was evaluated using observed agreement, Cohen’s kappa (κ) with 95% confidence intervals, and Gwet’s AC1. The diagnostic performance of ROSE, taking the pathologist’s adequacy assessment as the reference standard, was quantified in terms of sensitivity, specificity, positive predictive value (PPV), and negative predictive value (NPV), each with 95% confidence intervals. McNemar’s test was applied to assess asymmetry in discordant classifications. For final multicategory diagnoses, both κ and AC1 were calculated. Heat maps of contingency tables were generated to visualize agreement patterns. A two-sided p-value <0.05 was considered statistically significant.

## Results

A total of 78 patients were included (EBUS-TBNA: 37; cTBNA: 41). Mean age was 58.6 ± 14.1 years in the EBUS group and 60.1 ± 13.9 years in the cTBNA group.

### Technical adequacy

According to the pathologist team, all EBUS-TBNA samples were adequate (37/37, 100%, 95% CI 85.7–100) compared with 32/41 (78.0%, 95% CI 63.3–88.0) cTBNA samples. The difference was statistically significant (Fisher’s exact test p = 0.0026, OR = ∞ , 95% CI 2.05– ∞ ).

Agreement between the laboratory specialist (ROSE) and the team of pathologists for sample adequacy was high. The observed agreement reached 92.3%, with Cohen’s κ = 0.71 (95% CI 0.49–0.92), indicating substantial agreement, and Gwet’s AC1 = 0.90 (95% CI 0.81–0.98), corresponding to almost perfect agreement. Diagnostic performance was also excellent: sensitivity 91.3% (95% CI 82.3–95.9), specificity 100% (95% CI 70.1–100), positive predictive value (PPV) 100% (95% CI 94.2–100), and negative predictive value (NPV) 60.0% (95% CI 35.7–80.2). McNemar’s test confirmed a significant asymmetry in discordant cases (p = 0.041 with continuity correction; p = 0.014 without correction), suggesting that the laboratory specialist was more likely to classify samples as adequate when the team of pathologists did not.

**FIGURE 1:**
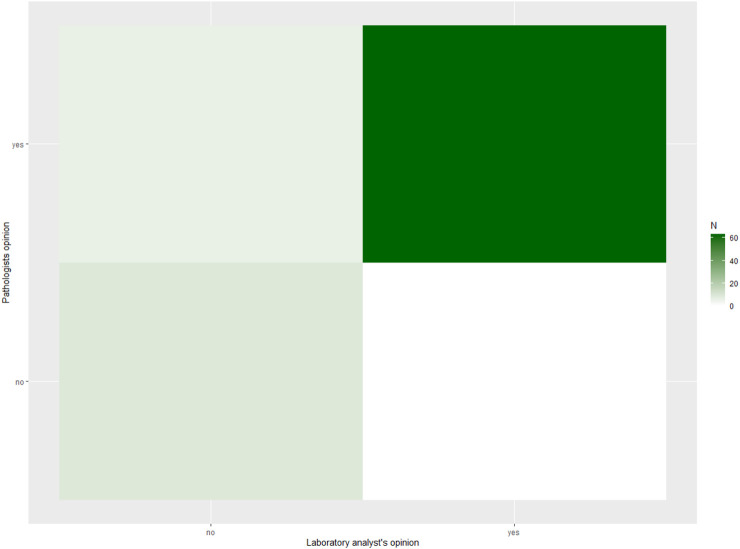
Heat map of ROSE versus pathologist adequacy assessment (n = 78). Heat map showing the contingency distribution between laboratory specialist (ROSE) and pathologist evaluations of sample adequacy (adequate vs. inadequate). Darker shading indicates higher case counts, and numbers within the cells represent the number of cases per category. Agreement was high, with most cases clustering in the “adequate/adequate” cell. Overall concordance was 92.3%, Cohen’s κ = 0.71 (95% CI 0.49–0.92), and Gwet’s AC1 = 0.90 (95% CI 0.81–0.98).

**FIGURE 2:**
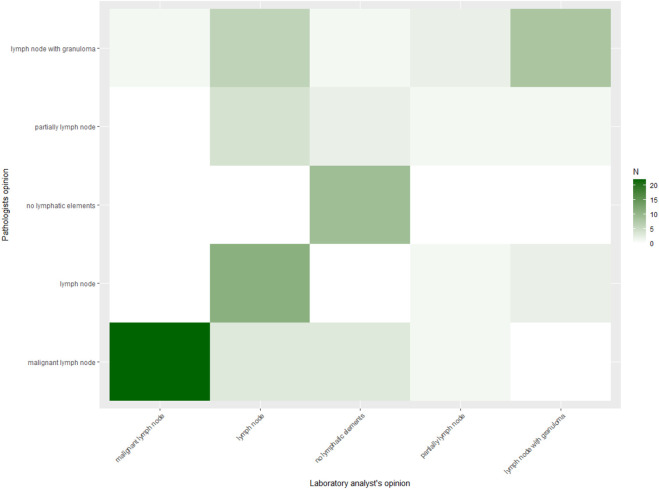
Heat map of ROSE versus pathologist final diagnosis (n = 78). Heat map displaying agreement between laboratory specialist and pathologist categorical final diagnoses. Cell counts are indicated numerically, with shading intensity proportional to frequency. Although concordance was substantial, discordant cases occurred across diagnostic categories. Agreement was 65.4%, Cohen’s κ = 0.55 (95% CI 0.42–0.68), and Gwet’s AC1 = 0.57 (95% CI 0.44–0.71).

**FIGURE 3:**
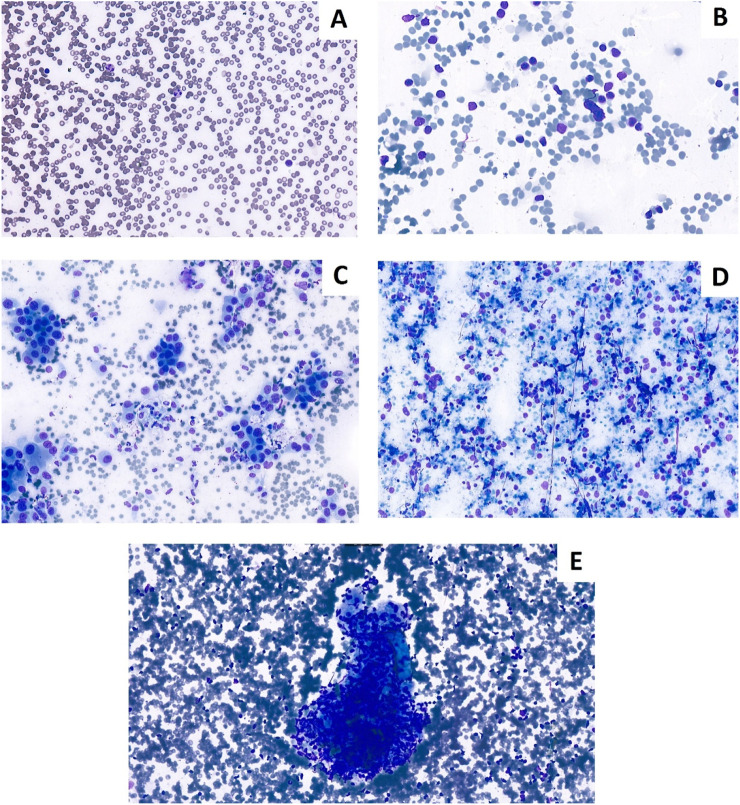
Cytological Diff-Quik stained smears prepared during bronchoscopy. **(A)** Non-representative smear for lymph node. **(B)** Representative lymph node smear. **(C)** Non-small cell lung cancer presentation lymph node smear. **(D)** Neuroendocrin carcinoma. **(E)** Granuloma in lymph node (Sarcoidosis).

In contrast, agreement on final categorical diagnosis was lower. The observed agreement was 65.4%, with Cohen’s κ = 0.55 (95% CI 0.42–0.68) and Gwet’s AC1 = 0.57 (95% CI 0.44–0.71), both consistent with moderate agreement. Heat maps (Figures 1, 2) illustrate these findings, with strong clustering in the “adequate/adequate” cell for adequacy assessment and a more dispersed distribution across categories for final diagnosis. Figure 3 shows characteristic cytological smear samples obtained during bronchoscopy.

## Discussion

In this study we evaluated the performance of rapid on-site evaluation (ROSE) performed by a university-trained laboratory specialist during bronchoscopic sampling, and compared the findings with the final cytopathologist report. Our results indicate that laboratory specialist–performed ROSE is highly reliable for the assessment of sample adequacy, with substantial-to-almost perfect agreement with the pathologists, while agreement on final diagnosis was moderate. This is especially important, as involvement of laboratory specialists could decrease the workload of pathologists, who are already missing from healthcare systems around the world [10, 11].

The adequacy results are particularly important from a clinical perspective. We observed 100% adequacy with EBUS-TBNA and 78% with cTBNA, consistent with previous studies demonstrating the superior technical yield of EBUS [3, 4]. The presence of a real-time adequacy assessment further enhances this efficiency by reducing the likelihood of nondiagnostic samples and minimizing the need for repeat procedures. ROSE performed by the laboratory specialist demonstrated high sensitivity (91.3%) and perfect specificity (100%) for adequacy, with an excellent positive predictive value. These results are similar, or better compared to previous studies, where ROSE was performed by a pulmonologist [12, 13]. These results confirm that adequacy can be reliably determined by trained personnel outside of pathology, offering a pragmatic solution where on-site cytopathology support is unavailable.

The moderate agreement on final diagnosis (κ = 0.55, AC1 = 0.57) is consistent with the intended role of ROSE. Similar findings have been reported in studies where pulmonologists or cytotechnologists performed ROSE: agreement with pathologists is typically excellent for adequacy but more limited for specific diagnostic categories [7–9]. ROSE is not designed to replace full cytopathologic evaluation, but to ensure that samples are adequate for subsequent processing and ancillary testing. The pattern of discordant cases in our analysis, including sarcoidosis and malignancy, reflects the inherent diagnostic limitations of on-site cytology without the use of ancillary stains and immunohistochemistry.

Our study contributes novel evidence by showing that a laboratory specialist (clinical biochemist) can effectively fulfill the role of on-site evaluator. This may be especially relevant in healthcare systems with limited cytopathology resources, where the increasing procedural workload makes it impractical to rely on pathologist presence during every bronchoscopy. Compared to solutions involving interventional pulmonologists performing ROSE themselves, which increases physician workload, involving trained laboratory staff provides a sustainable and scalable alternative.

Strengths of our study include the prospective collection of real-world data, application of multiple reliability indices (κ, AC1, diagnostic accuracy, McNemar’s test), and the direct comparison between EBUS and cTBNA. Limitations include the modest sample size, the single-center design, the lack of assessment of inter-observer variability, the lack of assessment of the impact on clinical practice, (such as the rate of repeat procedures or turnaround time), and the fact that only one laboratory specialist was evaluated, which may limit generalizability. Future research should include multi-center studies, training standardization, and assessment of the impact on clinical outcomes such as repeat procedure rates and time to diagnosis.

In conclusion, laboratory specialist–performed ROSE is a feasible and accurate alternative to pathologist on-site evaluation, ensuring high adequacy rates during bronchoscopic sampling. This approach can optimize diagnostic yield, support efficient patient management, and may help address resource limitations in interventional pulmonology and cytopathology.

## Data Availability

The raw data supporting the conclusions of this article will be made available by the authors, without undue reservation.
